# Understanding High-Risk Behavior in Mexican University Youth: Links Between Sexual Attitudes, Substance Use, and Mental Health

**DOI:** 10.3390/healthcare13121473

**Published:** 2025-06-19

**Authors:** Gustavo A. Hernández-Fuentes, Osiris G. Delgado-Enciso, Jessica C. Romero-Michel, Verónica M. Guzmán-Sandoval, Mario Del Toro-Equihua, José Guzmán-Esquivel, Gabriel Ceja-Espíritu, Mario Ramírez-Flores, Margarita L. Martinez-Fierro, Idalia Garza-Veloz, Fabian Rojas-Larios, Karla B. Carrazco-Peña, Rosa Tapia-Vargas, Ana C. Espíritu-Mojarro, Iván Delgado-Enciso

**Affiliations:** 1 Department of Molecular Medicine, School of Medicine, University of Colima, Colima 28040, Mexico; gahfuentes@gmail.com (G.A.H.-F.); 1933osiris@gmail.com (O.G.D.-E.); mequihua@ucol.mx (M.D.T.-E.); gcejae11@ucol.mx (G.C.-E.); mario_ramirez@ucol.mx (M.R.-F.); frojas@ucol.mx (F.R.-L.); dra_carrazco@ucol.mx (K.B.C.-P.); 2 State Cancerology Institute of Colima, Health Services of the Mexican Social Security Institute for Welfare (IMSS-BIENESTAR), Colima 28085, Mexico; 3 Faculty of Chemical Sciences, University of Colima, Coquimatlan 28400, Mexico; 4 Faculty of Law, University of Colima, Colima 28040, Mexico; jessica_romero@ucol.mx; 5 School of Psychology, University of Colima, Colima 28040, Mexico; gus_vero@ucol.mx; 6 Clinical Epidemiology Research Unit, Mexican Institute of Social Security, Villa de Alvarez, Colima 28984, Mexico; jose.esquivel@imss.gob.mx; 7 Molecular Medicine Laboratory, Unidad de Medicina Humana y Ciencias de la Salud, Universidad Autónoma de Zacatecas, Zacatecas 98160, Mexico; idaliagv@uaz.edu.mx; 8 Medical Education Auxiliary Coordination, Mexican Institute of Social Security (IMSS) OOAD Colima, Colima 28030, Mexico; rosa.tapia@imss.gob.mx; 9 Department of Pediatrics, Mexican Institute of Social Security (IMSS), General Hospital of Zone No. 1, Villa de Alvarez 28984, Mexico; aespiritu3@ucol.mx; 10 Robert Stempel College of Public Health and Social Work, Florida International University, Miami, FL 33199, USA

**Keywords:** erotophilia, mental health, substance use, sexual behaviors, university students

## Abstract

**Background/Objectives:** Sexual attitudes, particularly those on the erotophilia (positive openness) to erotophobia (negative fear) scales, play a critical role in shaping behaviors and health decisions. While associations between sexual behavior and substance use have been documented, limited research has explored how sexual attitudes relate to mental health and substance use among Latin American university populations. This study aimed to examine the associations among erotophilic attitudes, mental health symptoms (anxiety and depression), substance use risk, and sexual behaviors in Mexican university students. **Methods:** A cross-sectional observational study was conducted between 2019 and 2023 with 1475 undergraduate students aged 17–25 years. Participants completed the Revised Sexual Opinion Survey (R-SOS) to assess sexual attitudes, the Hospital Anxiety and Depression Scale (HADS) for mental health evaluation, and adapted items from the Alcohol, Smoking and Substance Involvement Screening Test (ASSIST) to measure substance use risk. Erotophilic attitudes were defined as R-SOS scores ≥ 70. Statistical tests included the Kolmogorov–Smirnov test for normality, *t*-tests or Mann–Whitney U tests for group comparisons, Fisher’s exact test for categorical variables, and Spearman’s correlations. Multivariate binary logistic regression was used to calculate adjusted odds ratios (AdORs) and 95% confidence intervals (CIs), with a significance level of *p* < 0.05. **Results:** Erotophilic students were more likely to be male, older, initiate sexual activity earlier, and report a greater number of sexual partners. Erotophilia was positively associated with anxiety and tobacco, alcohol and marijuana use, and negatively associated with depressive symptoms. Multivariate analysis indicated that erotophilia was independently associated with male sex, age ≥ 20, higher anxiety, lower depression, low socioeconomic status, and increased risk of tobacco and marijuana use. Lower rates of consistent condom use were also reported among erotophilic individuals. **Conclusions:** Erotophilia may serve as a behavioral risk marker linked to anxiety symptoms and increased substance use, but not to depression. These findings highlight the need for integrated interventions addressing sexual health, substance use, and mental well-being in university populations.

## 1. Introduction

Sexuality and substance use are central domains in the psychosocial development of university students, who often navigate the transition to adulthood within contexts marked by autonomy, experimentation, and identity formation [1]. Within this developmental framework, sexual attitudes—defined as stable affective-cognitive dispositions toward sexual stimuli—play a key role in shaping not only sexual behaviors but also broader lifestyle choices. Among these attitudes, the erotophilia–erotophobia continuum, introduced by Fisher and Byrne (1978), has proven particularly salient in understanding individual variability in sexual responses [2,3]. Erotophilia refers to a positive emotional and evaluative response to sexuality and sexual expression, whereas erotophobia is characterized by anxiety, guilt, or discomfort in reaction to sexual content or activity [4]. These attitudes are not merely personal preferences but are deeply rooted in sociocultural scripts, moral norms, and individual experiences.

Previous studies have shown that erotophilia is associated with increased sexual activity, a greater number of sexual partners, and more permissive sexual attitudes [5]. Notably, erotophilic tendencies may generalize to other risk-related domains, such as substance use, sensation-seeking, and disinhibited behavior [6,7]. In contrast, erotophobia has been linked to elevated anxiety and depressive symptoms, as well as reduced sexual satisfaction [8,9]. While a positive correlation between adolescent sexual activity and substance use has been reported [7], few studies have examined how sexual attitudes—independent of actual sexual behavior—relate to substance use. Furthermore, empirical research on these associations remains limited, particularly in Latin American university populations.

In recent years, global youth cultures have experienced significant shifts in attitudes toward sexuality, influenced in large part by the pervasive role of digital media. Social networks and online platforms such as Instagram, TikTok, OnlyFans, and dating applications (e.g., Tinder, Bumble) have contributed to the normalization of open sexual expression, the commodification of erotic content, and greater visibility of diverse sexual identities and practices [10,11]. These environments can foster erotophilic tendencies by promoting positive or curious attitudes toward sexuality, encouraging exploration, and reducing stigma [12,13]. At the same time, they may contribute to the pursuit of immediate gratification, exposure to risky behaviors, and distorted expectations of intimacy. Young people increasingly engage with sexual content online not only for educational or entertainment purposes but also as part of their identity construction and social belonging. This digital landscape plays a central role in shaping sexual attitudes and behaviors, often reinforcing hedonistic or disinhibited patterns [14]. Understanding erotophilia in contemporary youth thus requires considering how digital media ecosystems influence affective and behavioral responses to sexuality, particularly in contexts where sexual information is more accessible than ever but often lacks critical or health-informed perspectives [12,14].

Although Hispanic youth in the United States have been shown to exhibit higher lifetime rates of drug use and unprotected sexual activity compared to their non-Hispanic peers [15], few studies have focused on Latin American youth residing in their countries of origin. According to the 2019 National Survey on Drug, Alcohol and Tobacco Use (ENCODAT), approximately 17.2% of Mexican youth aged 18–25 report lifetime use of marijuana, while binge drinking is reported by 25.3% in the same age group [16]. Additionally, data from the 2021 National Health and Nutrition Survey (ENSANUT) indicate that consistent condom use among sexually active Mexican youth remains suboptimal, with only 54% reporting condom use during their last sexual encounter [17]. These statistics highlight the relevance of studying sexual attitudes, risk behaviors, and their mental health correlates in this population [1,18].

Understanding these relationships is critical in light of growing public health concerns, including rising rates of sexually transmitted infections (STIs), unintended pregnancies, and substance-related harm among young adults globally [19]. Meanwhile, evolving digital and social environments increasingly normalize both drug use and sexual experimentation, while many preventive strategies remain anchored in abstinence-based or risk-avoidance frameworks [20]. Considering the above, it is hypothesized that erotophilic attitudes will be positively associated with greater use of psychoactive substances, higher levels of depressive and anxiety symptoms, and increased engagement in risky sexual behaviors, such as inconsistent condom use and having multiple sexual partners. In contrast, erotophobic attitudes are expected to be associated with lower substance use and fewer sexual risk behaviors, potentially mediated by internalized sociocultural norms and higher emotional inhibition.

Accordingly, this study seeks to address these gaps by analyzing the interplay between erotophilic attitudes, substance use, mental health symptoms (depression and anxiety), and sexual behaviors—including condom use consistency and multiple sexual partnering—among university students in a region of Mexico. Through psychometric assessment and multivariate analysis, the study seeks to contribute a culturally grounded and integrative understanding of how sexuality, mental health, and risk behaviors intersect, and to inform the development of sex-positive, harm reduction-oriented interventions.

## 2. Materials and Methods

### 2.1. Study Design and Setting

This cross-sectional, observational study was conducted between 2019 and 2023 at the University of Colima, located in a city of approximately 400,000 inhabitants in western Mexico. Although cross-sectional in design, the study spanned nearly five years to ensure sufficient recruitment across different academic cohorts and to adapt to pandemic-related restrictions. Data collection occurred in three phases: in-person in 2019, online in 2021 (due to COVID-19 mobility limitations), and in-person again in 2023 after the return to normal academic operations. This extended timeline ensured representativeness across a changing educational and psychosocial context, particularly during and after the pandemic. The study analyzed different individuals during each time point, without following the same participants longitudinally. It was reported in accordance with the Strengthening the Reporting of Observational Studies in Epidemiology (STROBE) guidelines [21]. Its primary objective was to examine the association between sexual attitudes (erotophilia/erotophobia), mental health symptoms (anxiety and depression), and substance use behaviors among emerging adult university students enrolled in various undergraduate programs, including medicine, nursing, nutrition, law, mechatronics, architecture, and business administration.

### 2.2. Participants

Eligible participants were undergraduate students aged 17 to 25 years, actively enrolled in in-person educational programs. Recruitment was conducted through campus-wide announcements, online invitations, and in-class presentations. Exclusion criteria included a self-reported history of psychiatric disorders under treatment, cognitive impairment interfering with questionnaire comprehension, or incomplete data on key study variables. Students with a prior diagnosis of psychiatric illness were excluded to minimize confounding factors. Data was systematically screened for missing or anomalous entries. Participants with incomplete responses or inconsistent patterns were excluded. A non-probabilistic sampling method was employed, and a total of 1475 students completed the survey and were included in the final analysis. A total of 148 students either declined to participate in the study or failed to meet the inclusion criteria. 30 individuals were eliminated for having incomplete or inconsistent data. The survey was administered in three periods, in-person during school hours in 2019 and 2023, and online in 2021, reflecting adjustments to pandemic-related mobility restrictions and the subsequent resumption of normal academic activities. During the in-person phases, surveys were completed under research supervision, preventing participants from filling the survey more than once. For the online phase, invitations were distributed exclusively through each student’s unique institutional email address, which ensured that only one response per participant could be submitted. These measures were implemented to maintain the integrity of the data and avoid duplicate responses across the study period.

### 2.3. Ethical Considerations

The study was approved by the Research Ethics Committee of the State Cancer Institute and the University of Colima authorities (approval number CEICANCL230317-ASEXCCC-06, dated 2 March 2017). All participants provided written or electronic informed consent before enrollment, confirming the voluntary and anonymous nature of their participation. No identifying information was collected. The study adhered to the principles outlined in the Declaration of Helsinki and complied with all applicable local ethical standards.

### 2.4. Measures

#### 2.4.1. Sociodemographic Variables

Additional sociodemographic data were collected, including age, sex (male/female), employment status (currently employed or not), and weekly income, which was expressed using the Big Mac Index, whose unit represents the price of a Big Mac at McDonald’s in a given country and period [22]. The Big Mac Index also reflects disparities in currency valuations, cost of living, economic conditions, Big Mac affordability, and income inequality across regions [23,24,25,26]. Socioeconomic status, according to the 2018, or 2022 guidelines (for surveys conducted in 2019 or 2021/2023, respectively) of the Mexican Association of Market Intelligence and Opinion Agencies (AMAI, the Spanish acronym), considering D/E groups (vulnerable middle class/poor) as having a low socioeconomic level. Automobile ownership was also recorded, as it could be an indicator of socioeconomic status and has previously been shown to influence sexual behavior [1,27].

The rest of the variables like body mass index (BMI) were calculated based on self-reported height and weight. Self-esteem was assessed using the Rosenberg Self-Esteem Scale (RSES), a validated 10-item questionnaire, yielding scores ranging from 10 to 40, with higher scores indicating greater self-esteem. A score of 30 or more is considered high self-esteem [18,28].

#### 2.4.2. Sexual Attitudes

Sexual attitudes were evaluated using the Revised Sexual Opinion Survey (R-SOS), the Sexual Opinion Survey (SOS) was originally developed to measure the erotophobia–erotophilia dimension. A revised Spanish version (R-SOS) was later created to preserve the theoretical construct while adapting it linguistically and culturally for Spanish-speaking populations [4]. The R-SOS instrument is a validated 20-item that measures individual tendencies toward erotophilia (positive attitudes toward sexual stimuli) or erotophobia (negative attitudes). Responses were rated on a 7-point Likert scale ranging from 1 (strongly disagree) to 7 (strongly agree), yielding total scores between 20 and 140. Participants scoring ≥ 70 were classified as erotophilic, while those scoring ≤ 69 were classified as erotophobic, which is consistent with previous literature [4,29].

#### 2.4.3. Mental Health

Anxiety and depression symptoms were measured using the validated Spanish version of the Hospital Anxiety and Depression Scale (HADS). The HADS consists of 14 items, divided into two subscales, HADS-Anxiety (5 items) and HADS-Depression (6 items), with each item rated on a scale from 0 to 3. Subscale scores range from 0 to 21, with higher scores indicating greater symptom severity. Clinical cut-off points were defined as ≥8 for anxiety and ≥7 for depression [18]. Anxiety and depression statuses were categorized as co-variables.

#### 2.4.4. Sexual Behavior

Participants provided detailed information on their sexual behaviors, including the age at which they first engaged in vaginal or anal sexual intercourse, defined as penile penetration into the vagina or anus. They also reported the age at which they first participated in oral sexual activity. Additionally, participants indicated the total number of sexual intercourse partners they had throughout their lifetime, as well as the number of lifetime oral sex partners. To further explore the breadth of sexual experiences, participants specified the number of days since their last vaginal or anal sexual intercourse, as well as the number of days since their last oral sexual activity [1,30]. They were also asked whether they currently had a stable relationship. This information was collected given the recognized associations between sexual activity and mental health outcomes such as anxiety and depression during the emerging adulthood stage.

#### 2.4.5. Substance Use

Substance use behaviors were assessed through items adapted from the Alcohol, Smoking and Substance Involvement Screening Test (ASSIST). Participants reported both lifetime and current use of tobacco, alcohol, marijuana, cocaine/crack, amphetamines and other stimulants, inhalants, sedatives, hallucinogens, heroin/morphine, and other illicit drugs [31]. Patients were identified as being at moderate/high risk (moderate risk of health and other problems due to current substance use patterns; high risk are those subjects with a high probability of experiencing serious health, social, financial, legal, or relationship problems because of current use patterns and are likely to be dependent) [18].

### 2.5. Statistical Analysis

Data are presented as percentages, means with standard deviations, or medians with 25th and 75th percentiles for non-normally distributed variables. The normality of the data was assessed using the Kolmogorov–Smirnov test. Participants were dichotomized into two groups: those exhibiting erotophobic tendencies (R-SOS scores ≤ 69) and those with erotophilic tendencies (R-SOS scores ≥ 70). Variables with normal distribution were analyzed using the independent Student’s *t*-test for comparisons between two groups. For non-normally distributed variables, the Mann–Whitney U test was used. To compare qualitative data Fisher’s exact tests were used. To assess the relationship between R-SOS scores and other variables, Spearman’s correlation coefficients were calculated for continuous variables. To explore the associations between sexual attitudes, mental health (anxiety and depression), and substance use, multivariate binary logistic regression analysis was performed to determine adjusted odds ratios (AdORs) with their 95% confidence intervals (CIs) and *p*-values. The backward stepwise method was used for variable selection, with a *p*-value of 0.05 for entry and 0.15 for elimination.

Although participants were recruited during three distinct periods (pre-pandemic, during the pandemic, and post-pandemic), analyses were conducted on the entire sample as a whole. Importantly, the proportion of erotophilic and erotophobic individuals was relatively balanced across all three periods. This internal balance allowed comparisons without bias related to recruitment timing. Furthermore, the “study period” was included as a covariate in the multivariate models to statistically control for any potential confounding effects associated with the timing of data collection. Therefore, the possible influence of temporal factors on the associations of interest was minimized.

Receiver operating characteristic (ROC) curves were also generated to assess the discriminatory ability of significant variables. Statistical analyses were conducted using SPSS Statistics version 20 software (IBM Corp., Armonk, NY, USA) [32]. The required sample size was estimated using ClinCalc version 1 (https://clincalc.com/stats/Power.aspx; accessed on 10 May 2024) based on an anticipated small-to-medium effect size (Cohen’s d = 0.25), a significance level of 0.05, and a power of 0.90. The calculation suggested a minimum of 1036 participants. To account for incomplete responses and subgroup analyses, we aimed to recruit approximately 1500 students [33,34]. A *p*-value of <0.05 was considered statistically significant.

## 3. Results

Of the total participants, 57.15% reported having initiated sexual activity. Table 1 summarizes the general characteristics of the participants, stratified by their classification on the Revised Sexual Opinion Survey (R-SOS). Individuals with erotophilic tendencies (R-SOS score ≥ 70) were significantly more likely to be older (median age: 20 vs. 19 years; *p* < 0.001) and male (44.3% vs. 27.8%; *p* < 0.001) compared to their erotophobic counterparts (R-SOS score ≤ 69). A greater proportion of erotophilic participants had initiated sexual activity (67.8% vs. 46.9%; *p* < 0.001) and reported a higher number of sexual partners (median: 2 vs. 1; *p* < 0.001). Additionally, they reported an earlier initiation of oral sex (median age: 17 vs. 18 years; *p* < 0.001) and a higher number of oral sex partners (median: 2 vs. 1; *p* < 0.001). The proportion of students who reported having a stable partner or being employed did not differ significantly between groups. However, car ownership was significantly more common among erotophilic individuals. Interestingly, the time since the last sexual intercourse did not differ significantly between the groups.

### 3.1. Correlations Between Sexual Attitudes, Mental Health, and Substance Use

Table 2 presents the Pearson correlation coefficients between sexual attitudes (R-SOS scores), mental health indicators (HADS-A and HADS-D), and substance use (ASSIST scores). A significant negative correlation was found between R-SOS and depression scores (HADS-D; r = −0.094, *p* < 0.001), suggesting that higher levels of erotophilia are correlated with fewer depressive symptoms. However, no significant correlation was observed between R-SOS and anxiety scores (HADS-A; r = −0.038, *p* = 0.140).

In terms of substance use, R-SOS scores showed positive correlations with tobacco use (r = 0.125, *p* < 0.001), alcohol use (r = 0.155, *p* < 0.001), and marijuana use (r = 0.186, *p* < 0.001), indicating that individuals with more erotophilic attitudes tend to report higher levels of substance use. Additionally, R-SOS was positively correlated with the number of sexual partners (r = 0.263, *p* < 0.001) and negatively correlated with the age at first sexual intercourse (r = −0.135, *p* < 0.001), suggesting an earlier initiation of sexual activity among erotophilic individuals. Although several correlations in Table 2 reached statistical significance, it is important to note that most of these associations are relatively weak, as indicated by the low correlation coefficient values.

### 3.2. Substance Use Patterns by Sexual Attitude

With the aim of comparing substance use patterns between erotophobic and erotophilic individuals, Table 3 highlights significant differences in both lifetime use and current risk of substance use. Erotophilic participants reported higher lifetime use of tobacco (42.6% vs. 21.7%; *p* < 0.001), alcohol (85.0% vs. 71.0%; *p* < 0.001), and marijuana (34.3% vs. 12.8%; *p* < 0.001). They also exhibited higher rates of moderate to high risk for current use of these substances, as assessed by the ASSIST. Notably, erotophilic individuals had higher rates of moderate/high risk for the use of tobacco (28.7% vs. 14.7%; *p* < 0.001), alcohol (32.3% vs. 21.2%; *p* < 0.001), and marijuana (20.2% vs. 6.6%; *p* < 0.001).

### 3.3. Multivariate Analysis of Factors Associated with Erotophilic Tendencies

Table 4 presents the results of the multivariate logistic regression analysis identifying factors independently associated with erotophilic tendencies, defined as a Revised Sexual Opinion Survey (R-SOS) score ≥ 70. After adjusting for potential confounders, several sociodemographic, psychological, and behavioral factors emerged as significant.

Female participants exhibited lower odds of erotophilia compared to males (adjusted odds ratio [AdOR]: 0.493; 95% confidence interval [CI]: 0.377–0.645; *p* < 0.001). Being aged 20 years or older was associated with increased odds of erotophilia (AdOR: 1.681; 95% CI: 1.302–2.171; *p* = 0.001). Low socioeconomic status was inversely associated with erotophilia (AdOR: 0.657; 95% CI: 0.479–0.902; *p* = 0.009).

Psychological factors also played a significant role. Higher anxiety scores (HADS-A) were positively associated with erotophilic tendencies (AdOR: 1.428; 95% CI: 1.057–1.929; *p* = 0.020), whereas higher depression scores (HADS-D) were inversely associated (AdOR: 0.550; 95% CI: 0.411–0.734; *p* < 0.001).

Regarding moderate/high risk of substance use, current risk for marijuana use showed a strong positive association with erotophilia (AdOR: 3.785; 95% CI: 2.279–6.288; *p* < 0.001), and tobacco use was also associated (AdOR: 1.463; 95% CI: 1.014–2.112; *p* = 0.042). Interestingly, the use of heroin, morphine, or pain medications was inversely associated with erotophilic tendencies (AdOR: 0.238; 95% CI: 0.090–0.629; *p* = 0.004), suggesting a distinct pattern of substance involvement among less erotophilic individuals.

Because the study was conducted in 2019, 2021, and 2023 (pre-pandemic, during the COVID-19 pandemic emergency, and post-pandemic periods, respectively), we analyzed whether any of these periods was associated with a tendency toward erotophilia. Erotophilia was present in 50.6%, 49.7%, and 45.0% of participants in the three periods analyzed (2019, 2021, and 2023, respectively), with no significant differences between periods (*p* = 0.381, Fisher’s exact test). As shown in Table 4, none of the periods influenced the presence of erotophilia (OR 1.080, 95% CI 0.868–1.344; OR 1.032, 95% CI 0.841–1.266; and OR 0.818, 95% CI 0.610–1.096, respectively).

Table 5 examines whether the factors associated with erotophilic tendencies varied between students who had initiated sexual activity and those who had not. Among participants who had not yet engaged in sexual activity, factors with significant association of erotophilia included female sex (AdOR: 0.525; 95% CI: 0.347–0.795; *p* = 0.002), higher anxiety levels (AdOR: 1.619; 95% CI: 1.028–2.549; *p* = 0.038), lower depression levels (AdOR: 0.504; 95% CI: 0.328–0.775; *p* = 0.002), and marijuana use (AdOR: 3.982; 95% CI: 1.715–9.247; *p* = 0.001).

Among students who had initiated sexual activity, the pattern of factors with statistical relationship was generally consistent. Female sex (AdOR: 0.480; 95% CI: 0.336–0.687; *p* < 0.001), being over 20 years of age (AdOR: 1.725; 95% CI: 1.208–2.463; *p* = 0.003), low socioeconomic status (AdOR: 0.581; 95% CI: 0.391–0.863; *p* = 0.007), lower depression scores (AdOR: 0.700; 95% CI: 0.493–0.993; *p* = 0.046), and marijuana use (AdOR: 3.614; 95% CI: 2.104–6.208; *p* < 0.001) were all significantly associated with erotophilic tendencies. Notably, among sexually active participants, inhalant use was inversely associated with erotophilia (AdOR: 0.217; 95% CI: 0.064–0.734; *p* = 0.014).

These findings indicate that while certain associations—such as sex, mental health symptoms, and marijuana use—are consistent across groups, other factors, such as age and socioeconomic status, may play a more prominent role among those with sexual experience.

### 3.4. Associations Between Substance Use and Sexual Behaviors

Table 6 presents the associations between substance use and specific sexual behaviors. Consistent condom use was significantly less likely among females (AdOR: 0.556; 95% CI: 0.396–0.780; *p* = 0.001), individuals with high levels of erotophilia (AdOR: 0.687; 95% CI: 0.489–0.964; *p* = 0.030), and cannabis users (AdOR: 0.416; 95% CI: 0.282–0.613; *p* < 0.001). Having first sexual intercourse in an outdoor setting was associated with low socioeconomic status (AdOR: 2.495; 95% CI: 1.182–5.267; *p* = 0.016) and cocaine use (AdOR: 3.976; 95% CI: 1.472–10.737; *p* = 0.006).

The likelihood of having multiple sexual partners was positively associated with moderate to high risk related to the use of tobacco (AdOR: 2.218; 95% CI: 1.286–3.825; *p* = 0.004), cannabis (AdOR: 3.565; 95% CI: 1.989–6.389; *p* < 0.001), and amphetamines (AdOR: 6.030; 95% CI: 2.006–18.000; *p* < 0.001). Conversely, individuals at moderate to high risk due to hallucinogen use were significantly less likely to report multiple sexual partners during the same period (AdOR: 0.074; 95% CI: 0.017–0.333; *p* = 0.001). Being female was also associated with a decreased likelihood of reporting multiple concurrent sexual partners (AdOR: 0.294; 95% CI: 0.182–0.477; *p* < 0.001), but with increased odds of cohabiting with a partner (AdOR: 3.211; 95% CI: 1.052–9.802; *p* = 0.040). Furthermore, moderate to high risk related to tobacco use was strongly associated with cohabiting with a partner (AdOR: 5.077; 95% CI: 2.077–12.411; *p* < 0.001).

### 3.5. Significant Association of Sexual Attitudes for Substance Use Risk: ROC Analysis of Revised Sexual Opinion Survey (R-SOS)

The total score of the Revised Sexual Opinion Survey (R-SOS) demonstrated moderate predictive ability for cannabis use (AUC = 0.687, 95% CI: 0.650–0.725, *p* < 0.001), tobacco use (AUC = 0.621, 95% CI: 0.587–0.655, *p* < 0.001), and cocaine use (AUC = 0.622, 95% CI: 0.542–0.701, *p* = 0.005). Its predictive value was more limited for alcohol (AUC = 0.581), amphetamines (AUC = 0.581), hallucinogens (AUC = 0.565), and opioids (AUC = 0.553), with several of these results not reaching statistical significance (see Table 7).

Analysis at the item level revealed that certain R-SOS items were significantly associated with moderate to high risk use across multiple drug categories. Notably, Item 6 (“I am attracted by the idea of participating in a group sexual experience”) consistently demonstrated the highest discriminative accuracy, yielding an AUC of 0.673 (95% CI: 0.593–0.753, *p* < 0.001) for cocaine, 0.665 (95% CI: 0.623–0.707, *p* < 0.001) for cannabis, and 0.616 (95% CI: 0.535–0.696, *p* = 0.005) for amphetamines. Other items with meaningful predictive performance included Item 17 (“It would probably be an exciting experience to caress my own genitals”) and Item 16 (“It is very exciting to imagine uncommon sexual practices”), both of which yielded AUCs exceeding 0.60 for cannabis, cocaine, and hallucinogens.

In contrast, individual items demonstrated limited predictive utility for alcohol and opioid use, with most AUCs falling below 0.60 and many comparisons yielding non-significant results. Overall, these findings suggest that erotophilic sexual attitudes—particularly those reflecting openness to exploratory or non-normative sexual experiences—are more closely associated with the use of certain psychoactive substances, including cannabis, stimulants, and hallucinogens, than with substances considered more socially normative, such as alcohol or prescription opioids.

## 4. Discussion

This study offers critical insight into the psychosocial correlations of erotophilic attitudes among university students, highlighting their association with a broader spectrum of risk-related behaviors. In multivariable models, erotophilia emerged as a significant independent correlation of several risk profiles, particularly those involving substance use and sexual practices. Students with erotophilic orientations were more likely to engage in moderate to high risk use of substances, including cannabis (adjusted odds ratio [AdOR] 3.785; 95% CI 2.279–6.288) and tobacco (AdOR 1.463; 95% CI 1.014–2.112), compared to their erotophobic counterparts. Moreover, erotophilia was associated with higher prevalence of sexual activity with multiple partners and inconsistent condom use, reinforcing its linkage to pleasure-seeking and sensation-seeking tendencies. In contrast, an inverse association was observed between erotophilic attitudes and the use of heroin, morphine, or other opioid analgesics (AdOR 0.238; 95% CI 0.090–0.629), suggesting a differentiated risk profile in relation to substances more commonly associated with self-medication or emotional distress. These findings suggest that erotophilia may function as a psychological marker for engagement in a hedonistic behavioral repertoire, encompassing both sexual expression and psychoactive substance use, potentially shaped by normative, emotional, and contextual influences specific to young adult populations. These findings align with the previous literature that suggests that substance use is linked to increased sexual risk-taking; however, prior studies have primarily focused on behaviors (frequency of intercourse, number of sexual partners) rather than underlying sexual attitudes [7]. Schantz (2012) noted that while adolescent sexual practices correlate with substance use, other reports show that these associations do not necessarily imply causality [7,35]. Complex statistical models have indicated that the causal relationship between adolescent substance use and sexual activity may be less robust than traditionally reported. Notably, among males, alcohol and marijuana use have been shown to influence sexual activity, although cessation of substance use predominantly affected sexual behavior only for alcohol; for females, causal links were inconsistent [35].

Distinctively, the present study examines substance use within the context of sexual attitudes—specifically erotophilia—rather than over sexual behaviors. This distinction is critical in populations where sexual debut is delayed. Indeed, our findings indicate that more than 30% of individuals with erotophilic tendencies, and nearly half of individuals with tendencies erotophobic, had not yet initiated sexual intercourse (Table 1). Thus, erotophilia as measured here captures an emotional and attitudinal orientation towards sexuality that can exist independently of sexual experience [36]. From a psychosocial perspective, erotophilic individuals may display greater openness to sexual exploration and non-traditional scripts of intimacy, possibly facilitated by a liberal sociosexual orientation and reduced sexual guilt [37]. While such attitudes can support sexual agency and psychological well-being, they may also increase vulnerability to sexually transmitted infections (STIs) and unplanned pregnancies when not accompanied by consistent protective behaviors [37]. The relatively low rates of consistent condom use (67.0% vs. 74.6%; *p* = 0.018; see Table 1) and higher proportion of subjects with multiple sexual partners in this group (11.7% vs. 3.9%: *p* < 0.001; see Table 1) point to the ambivalence of erotophilic profiles: on the one hand, affirming sexual freedom and on the other, engaging in health-compromising behaviors.

When stratified by sexual experience, important differences emerge. Among students without sexual experience, erotophilia was associated with being male, having higher anxiety scores, lower depression scores, and marijuana use, while age and socioeconomic status were not significant factors. Among students who had initiated sexual activity, erotophilia was associated with being male, being 20 years or older, having higher socioeconomic status, lower depression scores, and marijuana use, with inhalant use being inversely associated with erotophilia.

These findings suggest that while gender, mental health indicators, and marijuana use are consistent factors with statistical relationship of erotophilia regardless of sexual experience, age and socioeconomic status become significant only among those who have initiated sexual activity. Furthermore, the negative association between inhalant use and erotophilia appears specific to sexually active individuals, indicating distinct substance use profiles depending on sexual behavior stages. Overall, erotophilia reflects an emotional and cognitive orientation toward sexuality that can precede sexual behavior [2], although its associations with substance use and mental health vary according to sexual experience.

These observations align with previous literature on sensation seeking and sexual risk-taking, where individuals who score high on measures of erotophilia and openness to experience are more likely to pursue novel or thrilling experiences, including experimentation with drugs and unprotected sex [38,39]. Alcohol and cannabis use in particular have been shown to mediate sexual disinhibition, reduce risk perception, and impair negotiation of safe sex practices [40], which may partially explain the co-occurrence of substance use and inconsistent condom use observed in this study [4].

Complementing these findings, receiver operating characteristic (ROC) curve analyses demonstrated that erotophilic attitudes, as measured by the Revised Sexual Opinion Survey (R-SOS), had a modest yet statistically significant association for identifying moderate or high risk of substance use, particularly for cannabis and cocaine. Items related to openness to unconventional or group sexual practices (e.g., Item 6) showed the highest individual predictive strength, suggesting that attraction to non-normative sexual experiences may reflect broader disinhibition or novelty-seeking tendencies. These tendencies may manifest in both sexual and drug-related behaviors.

The predictive strength was more robust for substances typically used in social or exploratory contexts, such as cannabis and cocaine, and weaker for substances like alcohol, opioids, or hallucinogens, whose use may be more contextually driven by psychosocial distress or cultural patterns. Item 6 (“I am attracted by the idea of participating in a group sexual experience”) consistently yielded the highest area under the curve (AUC) values across several substances, indicating its discriminative capacity. These results highlight the value of including sexuality-related psychological constructs in risk assessments and prevention programs for substance use, especially in youth populations where experimentation often blurs boundaries between sex and drug cultures.

In contrast, erotophobic attitudes have been associated with higher levels of anxiety and depression, lower substance use, and reduced sexual activity [41]. This pattern may reflect a more inhibited sexual identity shaped by sociocultural narratives that link sexuality with shame, guilt, or moral deviance. Erotophobia is often internalized through rigid gender norms, religious teachings, or family expectations, particularly in conservative contexts—leading to emotional conflict and psychological distress [42]. Although individuals with erotophobic attitudes may exhibit lower engagement in overt risk behaviors, the mental health consequences of sexual repression or avoidance can be significant, potentially manifesting as anxiety, somatization, or interpersonal difficulties. However, previous studies have also shown that anxiety can be a contributing factor to risk-taking behaviors, while depression has been linked to an increased number of sexual partners and higher substance use [43].

Anthropologically, these contrasting profiles underscore the ways in which sexual subjectivities are culturally constructed and differentially policed [44,45]. Erotophilic individuals may align with modern discourses of self-expression, body autonomy, and liberal sexuality, while erotophobic individuals may be more aligned with traditionalist frameworks that emphasize control, abstinence, and sexual normativity [46,47]. The presence of both profiles in the same student population highlights the cultural heterogeneity and tensions in contemporary sexual identities, shaped by competing narratives from media, religion, peer groups, and academic institutions. Additionally, digital environments such as social media platforms and dating apps may reinforce erotophilic tendencies by normalizing sexual exploration and amplifying exposure to both risk-promoting and risk-reducing messages, depending on the content consumed and peer interactions.

Importantly, the intersection of substance use with sexual behavior is not merely an individual choice but often embedded in social rituals and group dynamics. Alcohol and cannabis are commonly used as social lubricants or enhancers of sexual experience, particularly in university environments where experimentation is normalized and sometimes encouraged [48]. These practices may be interpreted as part of broader youth subcultures where sexual and pharmacological exploration are intertwined with identity formation, belonging, and pleasure-seeking.

The proportional representation of participants exhibiting erotophilic and erotophobic attitudes across all three data collection periods helped internally control for potential temporal effects. Having both cases (with erotophilia) and controls (without erotophilia) within each phase ensured comparability within each time point [49,50]. From an analytical perspective, this design minimizes confounding by time, as the associations between variables were assessed within a stable distribution of exposure status during each period. Additionally, to account for any residual effects related to the year of recruitment, “study period” was included as a covariate in the multivariate models. This strategy aligns with best practices in observational research, where internal comparability within strata is a recognized method to mitigate time-related bias [49,50]. Therefore, although some sample characteristics may have varied over time, the combination of internal balance and statistical adjustment reduces the likelihood of bias due to the timing of data collection.

From a public health standpoint, these findings suggest a need for integrated interventions that address the interconnections between sexuality, substance use, and mental health. Programs should go beyond abstinence messages or isolated risk prevention strategies. Instead, they should promote sex-positive education that empowers students to make informed, respectful, and safe choices [51,52], while also addressing the psychosocial functions of drug use and sexual behavior. Culturally sensitive frameworks that validate diverse sexual expressions and challenge stigma may be particularly effective in reducing internalized conflict and fostering mental well-being.

Limitations of this study include its cross-sectional design, which prevents the establishment of definitive causal relationships between the variables examined. Additionally, the reliance on self-reported data is a limitation, as participants may underreport or overreport certain behaviors, particularly those that are stigmatized, introducing potential bias. The study’s focus on students from a single university in Mexico restricts the generalizability of the findings to broader populations or different cultural settings. Despite these limitations, the study has several strengths. The use of validated instruments such as the Revised Sexual Opinion Survey (R-SOS), the Hospital Anxiety and Depression Scale (HADS), and an adapted version of the ASSIST enhances the reliability of the data collected. The large sample size of 1475 students contributes to the statistical power and robustness of the observed associations. Furthermore, the study addresses a crucial gap in research by exploring the interplay between sexual attitudes, mental health, and substance use specifically within a Latin American university population, providing valuable insights into a context less studied than Western settings. Finally, a potential limitation of this study is the increased risk of false positive findings due to multiple statistical tests conducted. Although several associations were statistically significant, multiple testing can inflate the likelihood of Type I errors. Future research should use longitudinal designs and objective measures to clarify causal links and reduce bias. Exploring gender and cultural differences is recommended. Erotophilia is associated with higher substance use and risky sex; erotophobia links to more anxiety and less substance use. Integrated health interventions are needed.

This study concludes that erotophilic attitudes in university students are significantly associated with a broader risk profile encompassing substance use and specific sexual practices. Erotophilia independently predicts moderate to high risk for cannabis and tobacco use and is associated with a higher likelihood of having multiple sexual partners and decreased consistent condom use. These findings suggest that erotophilia may act as a psychological marker for a hedonistic behavioral repertoire. Erotophobia is linked to higher anxiety and depression but lower engagement in substance use and sexual activity, highlighting contrasting psychosocial profiles. The results underscore the need for integrated public health interventions addressing sexuality, substance use, and mental health holistically.

## 5. Conclusions

This study reveals the coexistence of erotophobic and erotophilic profiles among Mexican university students, reflecting a tension between traditional values and more open attitudes toward sexuality. Notably, erotophilic profiles were more commonly associated with the use of psychoactive substances, suggesting a behavioral pattern linked to sensation-seeking or a lower perception of risk. In contrast, erotophobic individuals showed a lower prevalence of drug use, possibly due to a stronger internalization of restrictive social norms. However, given the extended data collection period spanning from 2019 to 2023, potential changes in attitudes and behaviors over time should be considered when interpreting these findings. Future research is needed to explore how sociocultural shifts and significant events—such as the COVID-19 pandemic—may influence these relationships over time. These findings highlight the importance of considering the psycho-emotional dimensions of sexuality when designing comprehensive prevention strategies targeting drug use among young university populations.

## Figures and Tables

**Table 1:** General characteristics of students according to their tendency toward erotophobia (score ≤ 69) or erotophilia (score ≥ 70) on Revised Sexual Opinion Survey (R-SOS).

Variable	Revised Sexual Opinion Survey (R-SOS)						
	Erotophobia (≤69) (*n* = 748)			Erotophilia (≥70) (*n* = 727)			*p*
	25	50	75	25	50	75	
Age (years) *	18.00	19.00	20.00	19.00	20.00	21.00	<0.001
Aged ≥ 20 years (%)		39.9%			54.8%		<0.001
Female (%)		72.2%			55.7%		<0.001
BMI *	20.70	23.04	25.72	21.22	23.43	26.33	0.008
Currently working (%)		22.6%			24.1%		0.498
Monthly income *	4.71	9.43	16.98	4.71	9.43	16.98	0.401
Low socioeconomic status (%)		22.3%			18.5%		0.074
Owns a car (%)		16.6%			21.2%		0.024
Grade average *	8.40	8.87	9.20	8.50	8.90	9.18	0.200
Rosenberg self-esteem scale *	25.00	30.00	34.00	26.00	31.00	35.00	0.055
Stable romantic relationship (%)		40.5%			41.1%		0.864
Multiple sexual partners (%)		3.9%			11.7%		<0.001
Has initiated sexual activity (%)		46.9%			67.8%		<0.001
Consistent condom use (%) ^a^		74.6%			67.0%		0.018
Age at first intercourse ^a^*	16.00	18.00	18.00	16.00	17.00	18.00	<0.001
Lifetime number of sexual partners ^a^*	1.00	1.00	3.00	1.00	2.00	4.00	<0.001
Time since last sexual intercourse (days) ^a^*	5.00	15.00	60.00	4.00	14.00	90.00	0.742
Age at first oral sex *	16.00	18.00	19.00	16.00	17.00	18.00	<0.001
Number of oral sex partners *	1.00	1.00	2.00	1.00	2.00	4.00	<0.001
Time since last oral sex (days) *	3.00	14.00	42.00	5.00	21.00	100.00	0.001
Time since last kiss (days) *	1.00	7.00	30.00	1.00	7.00	30.00	0.918
R-SOS score	44.00	54.00	61.00	77.00	84.00	94.00	<0.001
HADS-Anxiety	6.00	9.00	12.00	6.00	9.00	12.00	0.072
HADS-Depression	3.00	7.00	10.00	3.00	5.00	9.00	<0.001

* Values are presented as median (25th–75th percentile) or percentages, as appropriate. Variables marked with (a) include only participants who reported having initiated sexual activity. R-SOS: Revised Sexual Opinion Survey; BMI: Body Mass Index; IVSA: age of first sexual intercourse; No. Sexual Partners: Number of lifetime sexual partners; Time Since Last Intercourse: time (in months) since last sexual intercourse; Age 1st Oral Sex: age at first oral sex experience; No. Oral Sex Partners: number of lifetime oral sex partners; Time Since Last Oral Sex: time (in months) since last oral sex experience; Time Since Last Kiss: time (in months) since last romantic kiss; HADS-A: Hospital Anxiety and Depression Scale−Anxiety Subscale; HADS-D: Hospital Anxiety and Depression Scale–Depression Subscale.

**Table 2:** Correlation between sexual attitude scores, anxiety, depression, and substance use risk.

	R-SOS Score		HADS-A		HADS-D	
	r	*p*	r	*p*	r	*p*
R-SOS score	1.000		−0.038	0.140	−0.094 **	<0.001
HADS-A	−0.038	0.140	1.000		0.651 **	<0.001
HADS-D	−0.094 **	<0.001	0.651 **	<0.001	1.000	
Age at FSI	−0.135 **	0.000	−0.003	0.913	0.033	0.217
No sexual intercourse partners	0.263 **	0.000	−0.032	0.222	−0.018	0.488
Actual No partners	0.185 **	0.000	−0.016	0.767	−0.034	0.519
Days since last sexual intercourse	0.031	0.392	0.060	0.092	0.030	0.404
Age at first oral sex	−0.080 *	0.030	−0.100 **	0.007	−0.092 *	0.013
Number of partners (oral sex)	0.339 **	0.000	0.025	0.664	−0.043	0.451
Days since the last oral sexual intercourse	0.083 *	0.029	−0.008	0.826	−0.016	0.666
Time since last kiss (days)	0.022	0.459	0.085 **	0.005	0.021	0.481
Tobacco products	0.125 **	<0.001	0.009	0.736	0.011	0.687
Alcoholic beverages	0.155 **	<0.001	−0.026	0.324	−0.067 **	0.010
Marijuana	0.186 **	<0.001	0.080 **	0.002	0.076 **	0.004
Cocaine or crack	0.058 *	0.027	0.045	0.086	0.030	0.253
Amphetamines/stimulants	0.054 *	0.042	0.041	0.115	0.036	0.169
Inhalants	0.029	0.264	0.034	0.201	0.028	0.285
Sedatives/sleeping pills	0.050	0.060	0.193 **	<0.001	0.130 **	<0.001
Hallucinogens	0.034	0.195	0.049	0.064	0.042	0.107
Heroin/morphine/pain drugs	0.023	0.383	0.038	0.146	0.031	0.240
Others	0.021	0.417	0.064 *	0.015	0.063 *	0.016

R-SOS score: Revised Sexual Opinion Survey total score, measuring sexual openness. HADS-D: Hospital Anxiety and Depression Scale–Depression subscale score. Age at FSI: Age at first sexual intercourse. No sexual intercourse partners: Total number of lifetime sexual intercourse partners. Actual No partners: Current number of sexual partners. Age at first oral sex: Age when the participant first engaged in oral sex. Number of partners (oral sex): Total number of oral sex partners. Days since last oral sexual intercourse: Time since the last oral sex encounter (in days). Tobacco products (ASSIST): Risk level for tobacco product use based on the ASSIST score. Alcoholic beverages (ASSIST): Risk level for alcohol consumption based on the ASSIST score. Marijuana (ASSIST): Risk level for marijuana use based on the ASSIST score. Cocaine or crack (ASSIST): Risk level for cocaine or crack use based on the ASSIST score. Amphetamines/Stimulants (ASSIST): Risk level for stimulant drug use based on the ASSIST score. Sedatives/Sleeping pills (ASSIST): Risk level for sedative or sleeping pill use based on the ASSIST score. Spearman correlation coefficient values (ρ) are plotted. * Correlation is significant at 0.05 level (2-tailed). ** Correlation is significant at 0.01 level (2-tailed).

**Table 3:** Comparison between erotophobic (score ≤ 69) and erotophilic (score ≥ 70) groups based on Revised Sexual Opinion Survey (R-SOS) in relation to lifetime substance use and current moderate/high risk of use.

Substance	Lifetime Use			Moderate/High Risk for Current Use		
	Erotophobic	Erotophilic	*p*-Value	Erotophobic	Erotophilic	*p*-Value
Tobacco products	21.7%	42.6%	<0.001	14.7%	28.7%	<0.001
Alcoholic beverages	71.0%	85.0%	<0.001	21.2%	32.3%	<0.001
Marijuana	12.8%	34.3%	<0.001	6.6%	20.2%	<0.001
Cocaine or crack	4.0%	6.1%	0.044	2.5%	4.1%	0.102
Amphetamines/stimulants	3.8%	6.8%	0.013	3.0%	4.0%	0.388
Inhalants	3.0%	4.2%	0.258	2.3%	2.4%	0.999
Sedatives/sleeping pills	10.2%	13.6%	0.050	7.5%	9.2%	0.294
Hallucinogens	3.6%	5.2%	0.156	2.8%	3.3%	0.643
Heroin/morphine/pain drugs	2.9%	3.3%	0.760	2.3%	2.3%	0.999
Others	2.5%	3.7%	0.221	2.4%	2.3%	0.999
Illicit substances	18.5%	38.1%	<0.001	11.7%	24.7%	<0.001

Data are presented as percentages. Current risk was assessed using Alcohol, Smoking and Substance Involvement Screening Test (ASSIST). *p*-values correspond to comparisons between groups using Fisher exact tests.

**Table 4:** Multivariate logistic regression for detecting factors associated with erotophilic tendencies (Revised Sexual Opinion Survey ≥ 70).

Variable	Bivariate Model				Multivariate Model			
	OR	95% CI		*p*	AdOR	95% CI		*p*
		Lower	Upper			Lower	Upper	
Female	0.484	0.390	0.602	<0.001	0.493	0.377	0.645	<0.001
≥20 years old	1.832	1.482	2.263	<0.001	1.681	1.302	2.171	0.001
Lives with parents	1.044	0.910	1.198	0.542				
Owns a car	1.355	1.042	1.761	0.023				
Low socioeconomic status	0.788	0.607	1.023	0.073	0.657	0.479	0.902	0.009
Has a partner	1.024	0.818	1.281	0.837				
High self-esteem	1.131	0.922	1.388	0.237				
HADS-A	0.858	0.695	1.059	0.154	1.428	1.057	1.929	0.020
HADS-D	0.616	0.501	0.757	<0.001	0.550	0.411	0.734	<0.001
Tobacco products	2.324	1.790	3.017	<0.001	1.463	1.014	2.112	0.042
Alcoholic beverages	1.775	1.402	2.246	<0.001				
Marijuana	3.590	2.541	5.071	<0.001	3.785	2.279	6.288	<0.001
Cocaine or crack	1.690	0.930	3.071	0.085				
Amphetamines/stimulants	1.327	0.752	2.343	0.329				
Inhalants	1.033	0.523	2.041	0.925				
Sedatives/sleeping pills	1.241	0.853	1.805	0.260				
Hallucinogens	1.190	0.648	2.187	0.574				
Heroin/morphine/pain drugs	0.971	0.487	1.938	0.934	0.238	0.090	0.629	0.004
Others	0.963	0.483	1.921	0.915				
Survey periods								
Pre-pandemic	1.080	0.868	1.344	0.489				
Pandemic	1.032	0.841	1.266	0.766				
Post-pandemic	0.818	0.610	1.096	0.178				

Multivariate binary logistic regression was conducted to estimate adjusted odds ratios (AdORs) with corresponding 95% confidence intervals (CIs) and *p*-values. Variables included in the final, most parsimonious model were selected using a backward stepwise procedure, with entry and removal probabilities set at 0.15 and 0.25, respectively. Socioeconomic status was classified according to the guidelines of the Mexican Association of Market Intelligence and Public Opinion Agencies (AMAI) as follows: A/B (high), C (middle), and D/E (low: vulnerable middle class/poor). Mental health symptoms were assessed using the Hospital Anxiety and Depression Scale (HADS), with subscale scores ranging from 0 to 21. Clinical thresholds were defined as ≥8 for anxiety (HADS-A) and ≥7 for depression (HADS-D). The risk of substance-related problems was evaluated using the Alcohol, Smoking and Substance Involvement Screening Test (ASSIST), identifying individuals at moderate to high risk.

**Table 5:** Multivariate logistic regression models identifying factors associated with erotophilia (Revised Sexual Opinion Survey > 70), stratified by sexual experience.

Factors	Has Not Started Sexual Life				Has Started Sexual Life			
	AdOR	95% CI		*p*	AdOR	95% CI		*p*
		Lower	Upper			Lower	Upper	
Female	0.525	0.347	0.795	0.002	0.480	0.336	0.687	<0.001
≥20 years old					1.725	1.208	2.463	0.003
Low socioeconomic status					0.581	0.391	0.863	0.007
HADS-A	1.619	1.028	2.549	0.038				
HADS-D	0.504	0.328	0.775	0.002	0.700	0.493	0.993	0.046
Marijuana	3.982	1.715	9.247	0.001	3.614	2.104	6.208	<0.001
Inhalants					0.217	0.064	0.734	0.014

Adjusted odds ratios (AdORs), 95% confidence intervals (CIs), and *p*-values are presented for variables independently associated with erotophilia (R-SOS score > 60), stratified by whether participants had initiated sexual activity. Variables included in the models were selected using backward stepwise logistic regression. Socioeconomic status was categorized based on the Mexican Association of Market Intelligence and Public Opinion Agencies (AMAI) guidelines: A/B (high), C (middle), and D/E (low: vulnerable middle class/poor). The Hospital Anxiety and Depression Scale (HADS) was used to assess mental health symptoms, with subscale scores ranging from 0 to 21. Clinical thresholds were set at ≥8 for anxiety (HADS-A) and ≥7 for depression (HADS-D). Risk of substance-related problems was evaluated using the Alcohol, Smoking and Substance Involvement Screening Test (ASSIST), identifying moderate to high risk levels.

**Table 6:** Multivariate logistic regression identifying moderate/high risk by substance use and other factors associated with sexual behaviors in emerging adults.

Factors	Multivariate model			
	AdOR	95% CI		*p*
		Lower	Upper	
Consistent condom use				
Female	0.556	0.396	0.780	0.001
Erotophilic orientation	0.687	0.489	0.964	0.030
Cannabis use (mod/high risk)	0.416	0.282	0.613	<0.001
First sexual intercourse outdoors				
Low socioeconomic status	2.495	1.182	5.267	0.016
Cocaine use (mod/high risk)	3.976	1.472	10.737	0.006
First sexual intercourse in a car				
Amphetamine use (mod/high risk)	2.961	1.045	8.387	0.041
Multiple sexual partners				
Tobacco use (mod/high risk)	2.218	1.286	3.825	0.004
Cannabis use (mod/high risk)	3.565	1.989	6.389	<0.001
Amphetamine use (mod/high risk)	6.030	2.006	18.121	<0.001
Hallucinogen use (mod/high risk)	0.074	0.017	0.333	<0.001
Female sex	0.294	0.182	0.477	<0.001
Age ≥ 20 years	2.276	1.392	3.723	<0.001
Erotophilic orientation	1.754	1.032	2.982	0.038
Currently living with a partner				
Tobacco use (mod/high risk)	5.077	2.077	12.411	<0.001
Female sex	3.211	1.052	9.802	0.040
Age ≥ 20 years	5.214	1.513	17.966	0.009

Multivariate binary logistic regression models were applied to identify independent factors associated with various sexual behaviors in emerging adults, including consistent condom use, having first sexual intercourse outdoors or in a car, engaging with multiple sexual partners, and currently cohabiting with a partner. Adjusted odds ratios (AdORs), 95% confidence intervals (CIs), and *p*-values are reported. Variable selection was performed using backward stepwise regression with entry (*p* < 0.15) and removal (*p* > 0.25) criteria. Variables included in the initial models: anxiety (HADS-A ≥ 8), depression (HADS-D ≥ 7), high self-esteem (Rosenberg ≥ 30), moderate/high risk use of tobacco (≥4 points), alcohol (≥11 points), cannabis (≥4 points), cocaine (≥4 points), amphetamines (≥4 points), inhalants (≥4 points), sedatives or sleeping pills (≥4 points), hallucinogens (≥4 points), opioids (≥4 points), other substances (≥4 points), any illicit drugs; female sex; age ≥ 20 years; low socioeconomic status; erotophilic orientation (R-SOS > 70).

**Table 7:** Significant association (area under the curve) of individual Likert-scale R-SOS items and global score for identifying moderate/high risk substance use.

Substance	Variable	AUC	95% CI (Lower–Upper)	*p*-Value
Tobacco	Global	0.621	0.587–0.655	<0.001
	Item 6	0.612	0.577–0.648	<0.001
	Item 17	0.604	0.571–0.638	<0.001
	Item 4	0.588	0.554–0.622	<0.001
Alcohol	Global	0.581	0.548–0.613	<0.001
	Item 6	0.592	0.558–0.625	<0.001
	Item 1	0.57	0.537–0.602	<0.001
	Item 8	0.569	0.536–0.603	<0.001
Cannabis	Global	0.687	0.650–0.725	<0.001
	Item 6	0.665	0.623–0.707	<0.001
	Item 17	0.648	0.607–0.688	<0.001
	Item 16	0.631	0.588–0.674	<0.001
Cocaine	Global	0.622	0.542–0.701	0.005
	Item 6	0.673	0.593–0.753	<0.001
	Item 17	0.634	0.555–0.713	0.002
	Item 16	0.622	0.532–0.711	0.005
Amphetamines	Global	0.581	0.508–0.654	0.051
	Item 6	0.616	0.535–0.696	0.005
	Item 20	0.587	0.511–0.663	0.036
	Item 3	0.587	0.508–0.666	0.036
Hallucinogens	Global	0.565	0.480–0.650	0.144
	Item 16	0.64	0.551–0.728	0.002
	Item 17	0.607	0.521–0.693	0.017
	Item 6	0.597	0.511–0.683	0.03
Opioids	Global	0.553	0.458–0.648	0.296
	Item 6	0.597	0.497–0.696	0.057
	Item 17	0.583	0.488–0.679	0.101
	Item 16	0.577	0.474–0.680	0.13

The table includes the three R-SOS items with the highest significant association for each substance. Items are rated on a 7-point Likert scale. Items assessed: (1) “I think watching a movie or reading a book with erotic/sexual content could be entertaining”; (3) “Bathing naked with someone of the same or opposite sex could be an exciting experience”; (4) “Masturbation can be an exciting experience”; (6) “I am attracted by the idea of participating in a group sexual experience”; (8) “I would be sexually aroused by watching a sexually explicit movie”; (16) “It is very exciting to imagine uncommon sexual practices”; (17) “It would probably be an exciting experience to caress my own genitals”; (20) “I don’t dislike imagining that I am having sex with more than one person”.

## Data Availability

The original contributions presented in the study are included in the article; further inquiries can be directed to the corresponding authors.
